# A Happy Coincidence

**Published:** 1887-02

**Authors:** Clarence Willard Butler

**Affiliations:** Montclair, N. J.


### A HAPPY COINCIDENCE.

Clarence Willard Butler, M. D., Montclair, N. J.

"There is but one way to get rid of these troubles successfully, and that is by an operation that will paralyze the sphincter muscle long enough to allow the ulcer to heal. This can be done by forcible dilatation alone. I speak of old, well-developed fissures of the anus."

This positive statement, made on p. 306, Vol. IV (1886), of *The Homœopathic Journal of Obstetrics*, sets at rests forever the question of the curability of anal fissures by internal medication. It must, of course, from its *ex cathedra* character, be authoritative. It is not a statement of opinion, you will observe; it is a statement of positive fact. And so Lilienthal lays out an unnecessary amount of bootless labor for the poor doctor who knows no better when he sets him to study out the indications for twenty-four remedies for this disease; and Helmuth was talking "for buncome" only when he says that "fissures that have resisted other treatment for a considerable time," have been known to "yield readily to the action of Graph. or Nit. ac., especially the latter." Even Hughes shows a most unusual credulity when he cites cures of anal fissures as having been made by Drs. Marston and Allen with Graph. and Ratanhia respectively, while, as for the statements made of cures of this affection by internal medicaments, and these in high potencies, by Lippe, Dunham, Eggert, etc., why, the less said about the *reported* cures of such fossiliferous homœopaths, the better for modern progressive Homœopathy, and yet before this positive and authoritative statement of fact, I had in my ignorance attributed the disappearance of all signs of a fissure in the following case to the administration of the remedy, and had not the electric light of the above writer's positive knowledge entirely swallowed up my "tallow dip" of faith, I should have been tempted to depend upon the carefully selected homœopathic remedy for a cure of the next case of fissure which it may be my lot to treat. Now, under the bright effulgence of this illuminating beam, I shall, of course, have both thumbs in that coming rectum just as soon as I can get my patient drunk enough to allow it.

G. E., aged sixty years.—Short, fat, jolly, light complexion, and nervous temperament, consulted me January 16th, 1886, presenting the following history and symptoms: Life sedentary; habits convivial. Takes a drink of whisky or gin before breakfast, another before lunch, one on the way home from the office, another when he gets home just before dinner, then no more until bedtime unless he has callers in the evening, when he may take "a few drinks." Uses tobacco moderately. His bowels have been inclined to be loose for years. Is never constipated. Has *now* three or four passages daily; the first, attended with most severe cutting pain in the anus, is small, long, and of a brownish color; the subsequent stools are watery, and the call is so urgent that he only succeeds in getting to the closet by great haste; has once or twice miscalculated by a fraction of a minute with disastrous results. Pain *shooting up the rectum* with and after all stools, but especially after the first stool in the morning, which has consistency and form. The same pain at times while sitting, never while walking. Indeed, when the pain comes on, he can *relieve it by walking about*, and in this way only. Standing relieves for one moment only, then the pain returns.*

An examination reveals a fissure of the anus. The rectum was not examined, because any endeavor to introduce the speculum was attended with most excruciatingly painful spasm of the sphincter ani.

℞. Ignat. am.$^{34m}$ (Fincke, graft), one dose; Sac. lac.

January 25th (nine days later).—Reports that the pain was greatly relieved and growing better till two days ago. Had very little pain after stools and none between. For the last two days has grown steadily worse.

℞. Ignat. am.$^{34m}$. (F. graft) in water, twelve doses at intervals of two hours, then Sac. lac.

---

* These symptoms have been present for about six months, during which time he has used pile ointments, salves, washes, douches, etc., without benefit.

January 30th.—Better in all ways. Morning stool larger. Little and often no pain after stools, and none between. Sac. lac.

February 22d.—Meeting Mr. E. socially, I learned that he has not been aware of any trouble since a day or two after his last visit at my office. Considers himself well.

December 16th, 1886.—Remains well.

Now was not that a happy coincidence? The disappearance of the pain so shortly after the Ignatia, I refer to, of course. The *only* way to cure him was to paralyze his sphincter. His sphincter was not paralyzed. *Ergo*, he was not cured.

*Quod erat demonstrandum.* Mr. E., therefore, together with the patients of Drs. Allen, Marston, Lippe, Dunham, Eggert, etc., must go down to his grave with an uncured *fissura in ano*, but the bright ray that lightens in some measure this sad picture is that, though logically they must have the fissure, *they never know it.*

